# Religion, Social Connectedness, and Xenophobic Responses to Ebola

**DOI:** 10.3389/fpsyg.2021.678141

**Published:** 2021-07-12

**Authors:** Roxie Chuang, Kimin Eom, Heejung S. Kim

**Affiliations:** ^1^Department of Psychological and Brain Sciences, University of California, Santa Barbara, Santa Barbara, CA, United States; ^2^School of Social Sciences, Singapore Management University, Singapore, Singapore

**Keywords:** religion, collectivism, prejudice, xenophobia, culture

## Abstract

This study examined the role of religion in xenophobic responses to the threat of Ebola. Religious communities often offer members strong social ties and social support, which may help members cope with psychological and physical threat, including global threats like Ebola. Our analysis of a nationally representative sample in the U.S. (*N* = 1,000) found that overall, the more vulnerable to Ebola people felt, the more they exhibited xenophobic responses, but this relationship was moderated by importance of religion. Those who perceived religion as more important in their lives exhibited weaker xenophobic reactions than those who perceived religion as less important. Furthermore, social connectedness measured by collectivism explained the moderating role of religion, suggesting that higher collectivism associated with religion served as a psychological buffer. Religious people showed attenuated threat responses because they had a stronger social system that may offer resources for its members to cope with psychological and physical threats. The current research highlights that different cultural groups react to increased threats in divergent ways.

## Introduction

As globalization brings people closer, people experience more frequent and intensified global level threats. Shorter travel time for people also means diseases spread more quickly and widely; fear spreads even faster through the Internet. Not long ago, pandemic threats such as Ebola and Zika, and most recently COVID-19 stirred a considerable amount of fear and instigated potentially xenophobic psychological and behavioral reactions in the U.S. For example, athletes withdrew from the Olympics due to fear of contracting Zika (Palazzo, 2016), travel bans were proposed due to Ebola (Roberts, 2014) and COVID-19 has fueled anti-Asian sentiments globally (Human Rights Watch, 2020). Given that such responses to a pandemic are often ineffectual, and yet, pose societal and psychological costs, it is vital to understand psychological processes underlying people’s reactions to such pandemic threats.

One of the common responses to disease threats is xenophobia, especially when the disease originated from outgroups. Xenophobia is fear and hatred of strangers or foreigners (Merriam-Webster, 2019), represented by various components such as out-group rejection and ethnocentrism (e.g., Faulkner et al., 2004; Navarrete and Fessler, 2006). Past research has shown that perceived vulnerability to disease promotes in-group attraction, ethnocentrism, and out-group negativity (Navarrete and Fessler, 2006). Perceived threat of contagious disease also predicts fear and rejection of outgroup members, leading to more negative attitudes toward foreign peoples (Faulkner et al., 2004). In the Ebola outbreak, for example, strong xenophobic responses in the U.S. were documented, such as rejecting international students from countries with previous confirmed cases even after they were declared Ebola-free (Ahmed and Mendoza, 2014; Roberts, 2014).

Although xenophobia is a common response to threats (e.g., Faulkner et al., 2004; Navarrete and Fessler, 2006), research shows that people with different sociocultural backgrounds respond to a range of threats in divergent ways (e.g., Ko and Kim, 2010; Uskul and Over, 2014; Eom et al., 2016). In particular, individuals show distinct responses to threats depending on buffers and resources in the broader social and cultural context. For example, children of higher socioeconomic status (SES) experience less pronounced physiological responses and perceive lower levels of hostility in ambiguous interactions with potential social threat than do their lower SES counterparts (Chen and Matthews, 2001). That is, people show attenuated threat responses when their sociocultural contexts offer buffers against threats. Taking this perspective, the present research investigates one important aspect of people’s cultural affiliation—religion—which may provide social resources against threats. Specifically, this research examines how individual difference in personal importance of religion moderates xenophobic psychological reactions to a large societal threat in the context of Ebola outbreak in 2014.

Religion attenuates distress and defensiveness (e.g., Rogers, 1996; Seybold and Hill, 2001; George et al., 2002). For example, religious beliefs, measured by intrinsic religiousness, function as psychological buffers against existential threats, and experimental affirmation of religious beliefs decreases death–thought accessibility following mortality salience (Jonas and Fischer, 2006). Neural evidence also shows that stronger self-reported religious zeal and greater belief in God were associated with less distress and defensiveness reactions to errors (Inzlicht et al., 2009). For participants who believed in God, a prime with religious concepts lowered the amplitude of error-related negativity (ERN), which is associated with defensiveness and anxiety in response to errors. Religious primes had the opposite effect on those who did not believe in God (Inzlicht and Tullett, 2010).

These distress-reducing effects of religion could be explained by two psychological paths. One is through increasing or restoring the sense of control. Believing in a god who exerts control over the universe, or a religious doctrine that brings order to the world serves as a compensatory resource for control during times of low personal control (Kay et al., 2008). Believing that one shares responsibilities of solving a problem with God helps promote a sense of control in coping with stressful events (Pargament et al., 1998). Other correlational and experimental research also support the theory that religion promotes greater self-control (McCullough and Willoughby, 2009) and secondary control (Weisz et al., 1984), which allows people to adjust themselves and accept the situation to achieve a more desired goal.

Another path is through increasing the sense of community and belonging. The distress attenuating aspect of religion has been mainly explained by religious beliefs—specifically by belief in a supernatural agent such as God. However, religion is not only a cognitive belief system but also a social and community system. Religious communities offer members strong social ties and increased social support, which helps members cope with psychological and physical stress (Seybold and Hill, 2001; George et al., 2002; Wilson, 2005). Religious behavior is expected to mobilize greater cooperation and trust, and when threats to group survival are high, religious groups are expected to outlast others (Norenzayan and Shariff, 2008).

In the relevant literature, these two paths are considered by and large independently (but see Sasaki and Kim, 2011). However, findings from a study (Kim et al., 2016) that examined psychological responses to Ebola suggested that sense of belonging might be a precursor for sense of control, especially in the face of large societal level threats. The study found that when the threat of Ebola was perceived as high, people who felt stronger ties with their social groups (i.e., high collectivists) reported greater feelings of control over the situation and ability to protect themselves and own communities from the disease than those who do not feel as strong ties with their social groups (i.e., low collectivists).

Taken together, these findings suggest that people who are religious may feel a greater sense of control in the face of threat through social connection and sense of belonging, especially when the threat is beyond any individual’s control. Viewing oneself as a part of social group via religious affiliation would provide efficacy in threatening circumstance. The current research tested this idea by examining how people respond to perceived Ebola threat differently as a function of how much they consider religion to be an important part of their lives, and how this difference is explained by the sense of control afforded by social ties. In the present analysis, social ties were measured with participants’ responses to a collectivism scale. Collectivism, relative to individualism, prioritizes group goals over individual goals, and tends to foster less alienation and more social support (Triandis, 1989). The sense of strong social bond and interdependence that people have with their groups are at the core of collectivism (Triandis, 2001). Therefore, we use individuals’ collectivistic orientation as a measure of social ties. We examined xenophobic tendencies as the psychological response to the threat.

**FIGURE 1:**
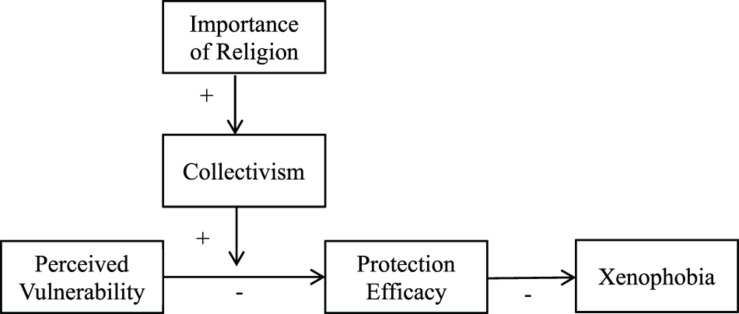
Theoretical model of mediated moderation of importance of religion.

In the present research, we reanalyzed a data set collected during the height of Ebola scare in 2014 (used in Kim et al., 2016). We hypothesized that importance of religion would moderate individuals’ responses to perceived threat of Ebola. The association between perceived vulnerability to Ebola and xenophobic responses would be weaker among people who perceived religion to be more important in their lives than those who perceived it as less important. We predicted that this moderation would be explained by higher collectivism among people who perceived greater importance of religion in their lives. We further theorize that this is because collectivism would provide people with efficacy to protect themselves and their communities from the threat of contagious disease. Thus, we examined protection efficacy from Ebola as a downstream psychological path leading to xenophobic responses from the moderating effect of religion importance. Our hypothesized model is shown in Figure 1.

## Methods

### Sample

A sample of *N* = 1000 that reflected US general population characteristics was constructed through YouGov (2015), an international research data group. YouGov used the full 2010 American Community Survey (Bureau, U. S. C., 2021), an annual demographics survey program conducted by the U.S. Census Bureau to gather information about the nation and its people, as a sampling frame. It matched respondents on gender (52% female), age (*M* = 46.46, *SD* = 17.06), race (70.3% White, 11.1% Black, 9.7% Hispanic, 4.8% Asian, 4.1% Other), education, region, political ideology, and political interest (for more detailed description of the sample, see Kim et al., 2016).

### Measures and Materials^1^

This study has received Institutional Review Board approval. Consenting participants completed an online survey on “Public Perception of Ebola” with items in the following order:

**Collectivism.** Collectivism was measured using eight items (e.g., “In the end, a person feels closest to members of his/her own religious, national, or ethnic group.”) adapted from Oyserman et al. (2002) (see also Triandis and Gelfand, 1998; Oyserman and Lauffer, 2002).^2^ Participants responded to how much they agreed or disagreed with the statements on a 7-point scale anchored at 1 (*strongly disagree*) and 7 (*strongly agree*) (*M* = 4.49, *SD* = 1.08, α = 0.81).

**Ebola information.** Participants read a passage about basic information on Ebola, adapted from the Centers for Disease Control and Prevention (2014b) web page. This step was included to ensure that participants were similarly informed about the disease. The passage provided factual information about Ebola, including the symptoms, cause, as well as history of the disease.

**Perceived vulnerability to Ebola.** To measure perceived risk of Ebola, the survey used nine questions adapted from the Perceived Risk of HIV Scale (Napper et al., 2012). These questions were divided into three sets: (1) perceptions of personal risk (e.g., “I feel vulnerable to Ebola infection”), (2) perceptions of local community risk (e.g., “I feel that people in my local community are vulnerable to Ebola infection”), and (3) perceptions of risk to country (e.g., “I feel that my country is vulnerable to outbreak of Ebola”). All items were assessed on 5-point scales anchored at 1 (*strongly disagree*) to 5 (*strongly agree*). The scores of all 3 sets were averaged to form a composite (*M* = 2.17, *SD* = 0.82, α = 0.91). These items were initially devised to capture potentially different functions of self, community, and country levels of vulnerability. However, analyses showed that they did not function meaningfully differently, and all 9 items formed a reliable measure. Thus, a composite of general perceived protection efficacy was created with 9 items.

**Perceived protection efficacy.** Perceived control in the context of Ebola was assessed with six items devised for the original study (Kim et al., 2016): two assessed perceived personal protection efficacy (e.g., “I feel confident that I can protect myself from Ebola”), two assessed community protection efficacy (e.g., “I feel confident that my local community can protect itself from Ebola”), and two assessed country protection efficacy (e.g., “I feel confident that my country can protect itself from Ebola”). All items were measured on 7-point scales anchored at 1 (*strongly disagree*) to 7 (*strongly agree*). The scores of 6 items were averaged to generate a composite (*M* = 4.63, *SD* = 1.21, α = 0.82). As with the perceived vulnerability measure, analyses showed that the self, community, and country protection efficacy did not function meaningfully differently, and all 6 items formed a reliable measure. Thus, a composite of general perceived protection efficacy was created with 6 items.

**Xenophobia.** Xenophobia is a multi-dimensional concept. In the existing literature, it has been operationalized as prejudice against outsider, ethnocentrism, and discriminatory actions (e.g. Faulkner et al., 2004; Navarrete and Fessler, 2006). In order to capture the multidimensionality of the concept, we assessed xenophobia with four elements, Two elements assessed outcomes directly related to Ebola: (1) prejudice toward West Africans and (2) support for restrictive travel policies. In addition to Ebola specific xenophobic measures, the other two elements assessed more generalized xenophobia toward outgroup members: (1) prejudice toward undocumented immigrants and (2) ethnocentrism.

To assess the prejudice toward West Africans and undocumented immigrants, participants rated their feelings toward the groups with 6 items, 3 were positive (e.g., acceptance, sympathy, and warmth) and 3 were negative (e.g., fear, disliking, and hostility). These items are a subset of the measure in Stephan et al. (1998). The scales ranged from 1 (*I do not feel this emotion at all*) to 8 (*I feel this emotion strongly*). Prejudice was the average of the negative minus the average of the positive items; higher scores indicated greater prejudice toward the groups (West Africans: *M* = −2.49, *SD* = 2.77, *a* = 0.72; undocumented immigrants: *M* = −0.87, *SD* = 3.45, *a* = 0.81 for composites based on target groups and valence). Participants then indicated their support for five restrictive policies related to Ebola, such as travel ban and quarantine (e.g., “A travel ban so that no planes can enter the United States from nations with high risk of Ebola” and “Mandatory 21-day quarantine for people coming from Liberia, Sierra Leone, Guinea”). They were given three choices: (1) “No, I would not sign the petition”; (2) “I support the policy, but do not wish to sign the petition”; or (3) “Yes, I would sign the petition in support of the policy” that formed the measure of policy support (*M* = 2.20, *SD* = 0.69, *a* = 0.91), with higher numbers indicating stronger support for restrictive policies. Ethnocentrism was assessed with two items from the American Ethnocentrism Scale (e.g., “People in the United States could learn a lot from people from other countries” and “Lifestyles in other countries are just as valid as in the United States.”; Neuliep and McCroskey, 1997). The scales ranged from 1 (*strongly disagree*) to 7 (strongly *agree*). Both items were reverse coded and averaged into a composite; higher number meaning more ethnocentric [*M* = 2.87, *SD* = 1.30; *r* (992) = 0.49, *p* < 0.001].

We created a latent variable of xenophobic tendency consisting of the four elements above. An initial exploratory factor analysis showed that the four components of xenophobia loaded onto a single factor (the eigenvalue of the first factor was 1.50, and all other factors had eigenvalues of less than one). This structure was replicated in a confirmatory factor analysis, which showed a one-factor model with a good fit (CFI = 0.99; RMSEA = 0.06; *χ*χ^2^(2) = 8.92, *p* = 0.012) (see also Kim et al., 2016 for more information on the construction of the latent variable). This latent variable served as our xenophobia outcome measure.

**Importance of religion.** Importance of religion was assessed with the question: “How important is religion in your life?” Participants responded on a 4-point scale anchored at 1 (*very important*) to 4 (*not at all important*). The scale was reverse coded, with higher scores meaning more importance of religion (*M* = 2.74, *SD* = 1.22).

**Demographic covariates.** Political ideology, gender (52% female), age (*M* = 46.46, *SD* = 17.06), education and income were controlled for in our analysis. Political ideology was assessed on a 5-point scale from 1 (*very liberal*) to 5 (*very conservative*). There were 91 participants who indicated “Not Sure,” which we assigned a 3 (*moderate*) score to for analytical purposes (*M* = 3.02, *SD* = 1.07). Education was measured with 5 categories (no high school: 6.3%, high school graduate: 37.6%, some college: 30.1%, 4 year college graduate: 17.4%, post graduate degree: 8.6%, median was some college). Annual family income was measured with 12 categories, from less than $10,000 to more than $150,000 per year (median was $40,000 – $49,999). (See Table 1 for bivariate correlations among all variables and Table 2 for partial correlations among key variables controlling for demographic covariates).

**TABLE 1:** Bivariate correlations among variables.

	**1.**	**2.**	**3.**	**4.**	**5.**	**6.**	**7.**	**8.**	**9.**
1. Perceived vulnerability	—								
2. Protection efficacy	−0.56***	—							
3. Collectivism	0.27***	−0.11***	—						
4. Importance of religion	0.26***	−0.22***	0.49***	—					
5. Xenophobia	0.40***	−0.39***	0.14***	0.23***	—				
6. Gender	0.04	−0.01	0.04	0.11***	−0.07*	—			
7. Age	−0.05	−0.01	0.15***	0.12***	0.09**	−0.001	—		
8. Education	−0.16***	0.17***	−0.05	−0.06	−0.24***	−0.02	0.06	—	
9. Income	−0.17***	0.12***	−0.04	−0.12***	−0.13***	−0.07*	0.11*	0.38***	—
10. Ideology	0.23***	−0.22***	0.18***	0.29***	0.58***	−0.02	0.05	−0.22***	−0.13***
*N* = 1000									

***p < 0.001, **p < 0.01, *p < 0.05.

**TABLE 2:** Partial correlations among vulnerability, efficacy, collectivism, importance of religion, and xenophobia with political ideology, age, gender, education and income as covariates.

	**1.**	**2.**	**3.**	**4.**
1. Perceived vulnerability	—			
2. Protection efficacy	−0.53***	—		
3. Collectivism	0.24***	−0.08*	—	
4. Importance of religion	0.18***	−0.16***	0.46***	—
5. Xenophobia	0.31***	−0.32***	0.01	0.07
*N* = 1000				

* p < 0<.05, ***p < 0<.001.

### Analytic Plan

We ran four structural equation models to test our hypotheses. Each model tested a key element of our overall model (Figure 1). We controlled for age, gender, income, education and political ideology in all analyses based on their significant correlations with either importance of religion or xenophobia, or with both. The main results remained consistent when these covariates were excluded. The results without the covariates are presented in Supplementary Material.

First, we ran a moderation analysis to examine the interaction between perceived vulnerability to Ebola and importance of religion on xenophobia. Second, after establishing the moderation, we tested whether collectivism underlies the moderating effect of importance of religion. We hypothesized that compared to individuals who perceive religion to be less important, people who perceive religion as more important show the weaker association between perceived vulnerability and xenophobia due to their greater collectivistic orientation. We adopted the mediated cultural moderation approach (e.g., Kim and Sherman, 2007; Uskul et al., 2009; Eom et al., 2018). This approach is an established form of mediated moderation analysis (Muller et al., 2005) to examine the psychological mechanisms behind moderation effects of culture variables. Specifically, it tests whether an observed cultural moderation is explained by a psychological factor(s) that is/are hypothesized to vary along the cultural moderator. Using this approach, we tested whether the moderation effect of importance of religion on the relationship between perceived vulnerability and xenophobia was explained by different levels of collectivism among less versus more religious people. Third, we examined protection efficacy as a proximal psychological mechanism leading to xenophobia by running a mediated moderation. We tested whether the interaction between perceived vulnerability to Ebola and importance of religion predicted xenophobia via protection efficacy. Finally, we tested the full model illustrated in Figure 1 by combining all the elements above.

## Results

### Moderation Analysis: Importance of Religion as Moderator

First, controlling for political ideology, gender, age, education, and income, we found a positive association between perceived vulnerability to Ebola threat and xenophobia, *r* = 0.31, *p* < 0.001 (without covariates : *r* = 0.40, *p* < 0.001). Then, we examined how importance of religion moderates the association between perceived vulnerability to Ebola and xenophobic tendencies. Importance of religion, perceived vulnerability, and their interaction were entered as predictor variables and xenophobia, the latent variable, was the outcome variable. Political ideology, gender, age, education, and income were also entered as covariates.^3^ We found that higher importance of religion predicted lower xenophobia (at the mean level of perceived vulnerability), β = −0.08, *b* = −0.05, *SE* = 0.03, 95% CI of *b* = [−0.10, −0.003], *p* = 0.037. Higher perceived vulnerability also significantly predicted greater xenophobia (at the mean level of importance of religion), β = 0.28, *b* = 0.30, *SE* = 0.04, 95% CI of *b* = [0.22, 0.38], *p* < 0.001.

**FIGURE 2:**
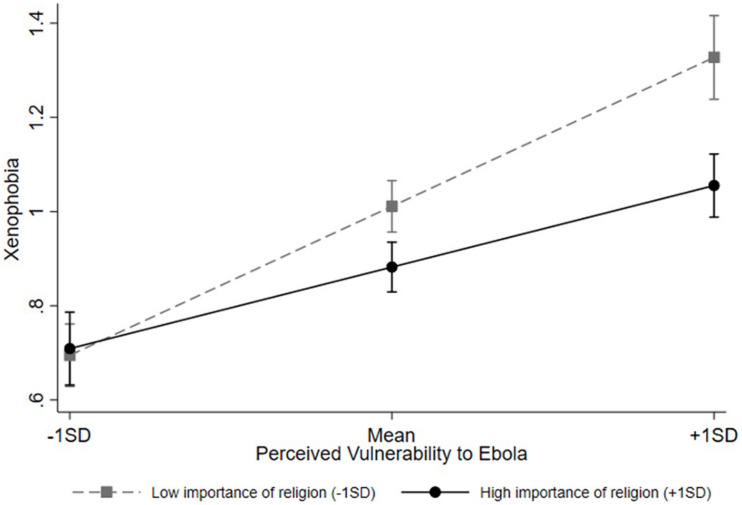
Interaction between perceived vulnerability to Ebola and importance of religion on xenophobia. Error bars indicate 95% confidence intervals.

Central to our key hypothesis, there was a significant interaction between perceived vulnerability and importance of religion, β = −0.08, *b* = −0.07, *SE* = 0.30, 95% CI of *b* = [−0.13, −0.01], *p* = 0.017. As Figure 2 illustrates, higher perceived vulnerability predicted higher xenophobia to a lesser extent among those with higher importance of religion (one standard deviation above the mean), β = 0.20, *b* = 0.21, *SE* = 0.05, 95% CI of *b* = [0.11, 0.31], *p* < 0.001, compared to those with lower importance of religion (one standard deviation below the mean), β = 0.36, *b* = 0.38, *SE* = 0.06, 95% CI of *b* = [0.27, 0.50], *p* < 0.001. Viewed differently, at lower levels of vulnerability (one standard deviation below the mean), there was no significant difference in xenophobia between participants with lower vs. higher importance of religion, β = 0.01, *b* = 0.01, *SE* = 0.03, 95% CI of *b* = [−0.06, 0.07], *p* = 0.890. However, at higher levels of vulnerability (one standard deviation above the mean), participants with higher importance of religion showed significantly lower xenophobic tendencies than those with lower importance of religion did, β = −0.16, *b* = −0.11, *SE* = 0.04, 95% CI of *b* = [−0.19, −0.04], *p* = 0.003.

### Mediated Cultural Moderation Analysis: Collectivism as Mediator

To examine the mediating role of collectivism, we conducted a mediated cultural moderation analysis, controlling for the same variables as the previous analysis (following the procedure outlined in Muller et al., 2005). Through the series of analysis, we tested whether collectivism varies along importance of religion, and collectivism mediates the moderating effect of importance of religion on the association between perceived vulnerability and xenophobia. The critical part of this analysis was to enter two interactions terms: (1) perceived vulnerability by importance of religion and (2) perceived vulnerability by collectivism on xenophobia simultaneously in the model. In so doing, we examine whether the original interaction involving importance of religion becomes non-significant or weaker, but the interaction involving collectivism significantly predicts xenophobia indicating mediated moderation.

The results of the series of SEM are presented in Table 3. First, we examined how vulnerability, importance of religion, and their interaction predicted xenophobia. This analysis is equivalent to the moderation results reported above and depicted in Figure 2. Consistently, importance of religion significantly moderated the association between perceived vulnerability and xenophobia. In the second equation, collectivism was entered as the outcome predicted by vulnerability, importance of religion, and their interaction. As predicted, there was a main effect of importance of religion, β = 0.43, *b* = 0.39, *SE* = 0.03, 95% CI of *b* = [0.33, 0.44], *p* < 0.001. More religious individuals showed stronger collectivism. There was also a main effect of vulnerability, β = 0.18, *b* = 0.24, *SE* = 0.04, 95% CI of *b* = [0.16, 0.31], *p* < 0.001. Finally, in the third equation, we entered vulnerability, importance of religion, vulnerability by importance of religion interaction, collectivism, and vulnerability by collectivism interaction as predictors and xenophobia as the outcome variable. The results indicated that the vulnerability by collectivism interaction was significant, β = −0.09, *b* = −0.08, *SE* = 0.03, 95% CI of *b* = [−0.14, −0.02], *p* = 0.011. Specifically, higher perceived vulnerability predicted higher xenophobia to a lesser extent among those with higher collectivism (one standard deviation above the mean), β = 0.23, *b* = 0.24, *SE* = 0.05, 95% CI of *b* = [0.14, 0.33], *p* < 0.001, compared to those with lower collectivism (one standard deviation below the mean), β = 0.38, *b* = 0.41, *SE* = 0.06, 95% CI of *b* = [0.29, 0.52], *p* < 0.001. In contrast, the vulnerability by importance of religion interaction was non-significant, β = −0.05, *b* = −0.04, *SE* = 0.03, 95% CI of *b* = [−0.10, 0.02], *p* = 0.195. Thus, the interaction between vulnerability and collectivism mediated the interaction between vulnerability and importance of religion on xenophobia, showing that collectivism mediated the moderating effect of importance of religion on the relationship between vulnerability and xenophobia.

**TABLE 3:** SEM results for mediated cultural moderation.

	**SEM 1 criterion: Xenophobia**		**SEM 2 criterion: Collectivism**		**SEM 3 criterion: Xenophobia**	
**Predictor**	***β (b)***	***z***	***β (b)***	***z***	***β (b)***	***z***
Vulnerability	0.28 (0.30)	7.30***	0.18 (0.24)	5.83***	0.30 (0.32)	7.65***
Importance of Religion	−0.08 (−0.05)	−2.09*	0.43 (0.39)	13.69***	−0.07 (−0.05)	−1.76
Vulnerability X Importance of Religion	−0.08 (−0.07)	−2.38*	−0.05 (−0.06)	−1.73	−0.05 (−0.04)	−1.29
Collectivism					−0.03 (−0.02)	−0.72
Vulnerability X Collectivism					−0.09 (−0.08)	−2.55*

*p < 0.05, ***p < 0.001 (following the procedure outlined in Muller et al., 2005; Kim and Sherman, 2007).

### Mediated Moderation Analysis: Protection Efficacy as Mediator

We tested whether the vulnerability by importance of religion interaction on xenophobia was mediated by protection efficacy. Importance of religion, perceived vulnerability, and their interaction were entered as predictor variables. Protection efficacy was the proposed mediator, and xenophobia, the latent variable, was the outcome variable. We controlled for the same variables as the previous analysis. The results indicated that the interaction between perceived vulnerability and importance of religion significantly predicted protection efficacy, β = 0.07, *b* = 0.09, *SE* = 0.04, 95% CI of *b* = [0.02, 0.16], *p* = 0.014. Specifically, the negative association between perceived vulnerability and protection efficacy was weaker among those with higher importance of religion (one standard deviation above the mean), β = −0.48, *b* = −0.69, *SE* = 0.06, 95% CI of *b* = [−0.80, −0.58], *p* < 0.001, compared to those with lower importance of religion (one standard deviation below the mean), β = −0.63, *b* = −0.90, *SE* = 0.06, 95% CI of *b* = [−1.02, −0.77], *p* < 0.001. Protection efficacy in turn negatively predicted xenophobic responses, β = −0.21, *b* = −0.15, *SE* = 0.03, 95% CI of *b* = [−0.21, −0.09], *p* < 0.001. The indirect effect of interaction between perceived vulnerability and importance of religion on xenophobia via protection efficacy was significant, β = −0.02, *b* = −0.01, *SE* = 0.01, 95% CI of *b* = [−0.02, −0.002], *p* = 0.026, and the direct effect was also significant, β = −0.07, *b* = −0.06, *SE* = 0.03, 95% CI of *b* = [−0.12, −0.003], *p* = 0.039. Thus, protection efficacy partially mediated the effect of the importance of religion by perceived vulnerability on xenophobia. Direct effect of vulnerability on xenophobia, β = 0.17, *b* = 0.18, *SE* = 0.04 95% CI of *b* = [0.10, 0.27], *p* < 0.001, and importance of religion on xenophobia, β = −0.08, *b* = −0.06, *SE* = 0.03, 95% CI of *b* = [−0.11, −0.01], *p* = 0.020, were significant.

### SEM Analysis

Lastly, we ran an extended SEM to test the full model in Figure 1. Political ideology, gender, age, education, and income were also entered as covariates for xenophobia. Results showed an acceptable fit: comparative fit index (CFI) = 0.86, root-mean-square error of approximation (RMSEA) = 0.08, χ^2^(48) = 296.84, standardized root-mean-square-residual (SRMR) = 0.05.^4^ The results of individual paths were also consistent with our hypothesized model. First, importance of religion was a significant predictor of collectivism; greater importance of religion predicted higher collectivism, β = 0.49, *b* = 0.43, *SE* = 0.03, 95% CI of *b* = [0.38, 0.49], *p* < 0.001. Collectivism in turn moderated the association between perceived vulnerability and xenophobia via both direct and indirect paths. The direct path of the interaction between vulnerability and collectivism on xenophobia was significant, β = −0.07, *b* = −0.06, *SE* = 0.03, 95% CI of *b* = [−0.12, −0.001], *p* = 0.045. Consistent with the results in the mediated moderation with collectivism as mediator above, higher perceived vulnerability predicted higher xenophobia to a lesser extent among those with higher collectivism (one standard deviation above the mean), β = 0.13, *b* = 0.14, *SE* = 0.05, 95% CI of *b* = [0.04, 0.24], *p* = 0.005, compared to those with lower collectivism (one standard deviation below the mean), β = 0.26, *b* = 0.27, *SE* = 0.06, 95% CI of *b* = [0.15, 0.39], *p* < 0.001.

**FIGURE 3:**
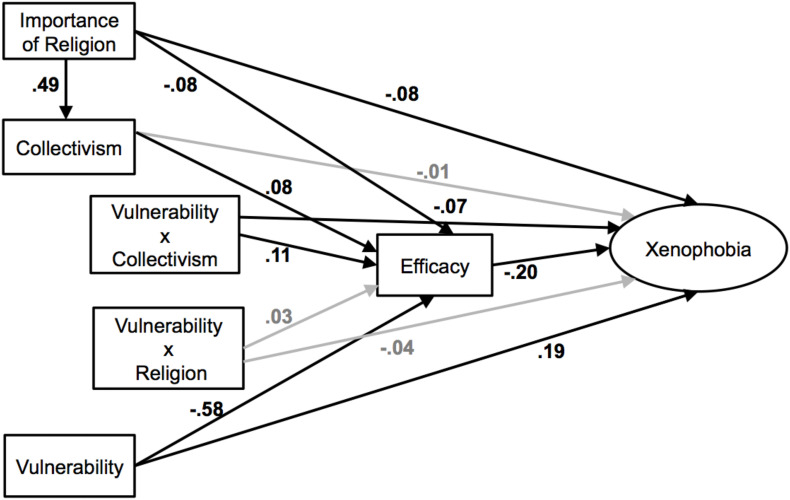
Structural equation model examining whether importance of religion moderates the association between perceived vulnerability and xenophobia through collectivism. The values shown are standardized path coefficients; black lines represent significant paths and gray lines represent non-significant paths (*p* > 0.05).

The indirect path of the vulnerability X collectivism interaction through protection efficacy was also significant, β = −0.02, *b* = −0.02, *SE* = 0.01, 95% CI of *b* = [−0.03, −0.01], *p* = 0.003. Specifically, the interaction between perceived vulnerability and collectivism on protection efficacy was significant, β = 0.11, *b* = 0.13, *SE* = 0.04, 95% CI of *b* = [0.06, 0.21], *p* < 0.001; negative association between perceived vulnerability and protection efficacy was weaker among those with higher collectivism (one standard deviation above the mean), β = −0.48, *b* = −0.70, *SE* = 0.05, 95% CI of *b* = [−0.81, −0.60], *p* < 0.001, compared to those with lower collectivism (one standard deviation below the mean), β = −0.68, *b* = −0.99, *SE* = 0.06, 95% CI of *b* = [−1.12, −0.87], *p* < 0.001. In turn, lower protection efficacy predicted greater levels of xenophobia, β = −0.20, *b* = −0.14, *SE* = 0.03, 95% CI of *b* = [−0.20, −0.08], *p* < 0.001. The original interaction between vulnerability and importance of religion was non-significant in predicting both protection efficacy and xenophobia in this model in which the interaction between vulnerability and collectivism was included (β = 0.03, *b* = 0.04, *SE* = 0.04, 95% CI of *b* = [−0.03, 0.11], *p* = 0.301 in predicting protection efficacy; β = −0.04, *b* = −0.04, *SE* = 0.03, 95% CI of *b* = [−0.10, 0.02], *p* = 0.234 in predicting xenophobia). See Figure 3 for SEM model.

## Discussion

The present study found that the tendency to be more xenophobic in response to pandemic threat such as Ebola outbreak significantly depends on people’s perceived importance of religion. Although participants generally were more xenophobic when they perceived themselves to be more vulnerable to Ebola, the association between the vulnerability perception and xenophobic tendencies was weaker among highly religious people. That is, individuals who felt religion is an important part of their lives showed weaker xenophobic reactivity to perceived vulnerability. These attenuated threat responses were associated with a stronger sense of social ties (i.e., high collectivism), which seems to offer greater control and efficacy over the pandemic threat to people for whom religion is important.

Research has often showed that religion increases negative attitudes and aggression toward outgroup members (Ginges et al., 2009; Johnson et al., 2012; LaBouff et al., 2012) and that the prosociality promoted by religion is limited only to members of the religious ingroup (Saroglou, 2006; Pichon and Saroglou, 2009). At the same time, previous research also showed that the relationship between religiosity (general religiousness or religious ideology) and prejudice may become non-significant once political ideology was included as a mediator (Laythe et al., 2001; Rowatt, 2018). Reflecting the complexity in this literature, we did find a positive correlation between importance of religion and xenophobia that goes away once controlled for political ideology, age, gender, income and education. Although the direct link between religion and xenophobia is not the focus in the present research, the present results continue to question whether religion itself is a key factor that brings about xenophobic responses. Regardless of the nature of the direct relationship between religiosity and xenophobia, our research focuses on how importance of religion may moderate people’s responses to perceived disease threat. Being highly religious may serve as a social buffer against societal threats such as Ebola outbreak, mitigating negative psychological reactions to threats, such as xenophobia that aggravate conflicts and divides in society. Specifically, the strong sense of social affiliation rooted in religious people affords greater efficacy over threat. Consequently, when facing societal issues such as Ebola, religious people may be less likely to convert their feelings of vulnerability into hostility toward outgroup members.

Because the present study relies on correlational design, we cannot draw causal conclusions. In addition, it is important to note that our key moderator, importance of religion, was measured with a single item. Previous research shows that single-item religiosity measures are common and effective (e.g., Norenzayan and Hansen, 2006; Gebauer et al., 2013), and thus, we believe that the single-item is a valid measure of the key concept. Nevertheless, we recognize that it does not fully capture the complexity of religion or religiosity. Future research should employ measures that can capture the roles of multiple facets of religiosity. Any forms or aspects of religiosity (not only importance of religion) that offers social ties and belongingness may provide similar buffering functions. In addition, different dimensions of religiosity may be associated with varying attitudes and behaviors. For example, intrinsic religiosity was positively correlated with altruism and negatively correlated with intergroup hostility, while extrinsic religiosity was uncorrelated or negatively correlated with altruism (Chau et al., 1990; Golec de Zavala et al., 2012). Consideration of this complexity of religiosity as a concept in future work is necessary to uncover which aspect of being religious garners the threat buffering effects and which aspect heightens xenophobic tendencies and outgroup hostility.

The present findings suggest that religious individuals gain a sense of control and protection through strong social affiliation. Perhaps religion encourages secondary control tendencies for those who are typically not as inclined to exercise secondary control, for example, European Americans who are from cultural contexts that emphasize personal agency and primary control (e.g., Morling et al., 2002, 2003). This increase in exercising secondary control may be the key to allowing people to be more integrated in their community and enjoy a greater sense of affiliation (Sasaki and Kim, 2011). However, it is important to note that in addition to collectivism, there could be other factors, such as pro-sociality and reduced hostility, that may explain the moderating role of religiosity. Past research has demonstrated an association between religion and pro-sociality. For example, when primed with religious words, people cheated less in subsequent tasks (Randolph-Send and Nielsen, 2007). Another study found that people allocated more money to strangers in an anonymous dictator game when God (compared to neutral or no) concept was implicitly activated (Shariff and Norenzayan, 2007). Research has also found that when primed with general religion belief system (“Which religious belief system do you identify with?”), people responded to a threat with reduced hostility in thoughts, behaviors, and judgements (Schumann et al., 2014). Perhaps importance of religion attenuated people xenophobic threat responses through increased pro-sociality and reduced hostility. Future research can directly investigate these other specific processes.

Although the data collected for this study is specific to a disease threat, the implications of our findings may not be confined to the particular issue of disease responses. For example, being religious may also shape people’s responses to other social issues that bring fear and threats in society, such as terrorism, inflow of refugees, and climate change (e.g., Eom et al., 2021). Responding to these issues with disproportionate fear and xenophobia involves significant social costs. Researching the ways religion modulates psychological reactions to social challenges would offer the invaluable knowledge and insights to reduce the costly responses of citizens in society.

## Data Availability Statement

The datasets presented in this study can be found in online repositories. The names of the repository/repositories and accession number(s) can be found below: https://osf.io/84zg3/.

## Ethics Statement

The studies involving human participants were reviewed and approved by UCSB Office of Research Institutional Review Board. Written informed consent for participation was not required for this study in accordance with the national legislation and the institutional requirements.

## Author Contributions

All authors jointly developed the ideas presented in this article. HK designed the study and collected the data. RC and KE analyzed the data. All authors drafted the article, provided critical revisions, and approved the final version of the manuscript for submission.

## Conflict of Interest

The authors declare that the research was conducted in the absence of any commercial or financial relationships that could be construed as a potential conflict of interest.

## Footnotes

1 We included all variables that are conceptually relevant to responses to Ebola threat in this analysis. The original dataset also included scales related to personal health symptoms and well-being, which were not theoretically related to this paper, therefore, not considered in our analysis. All data and materials used in Kim et al. (2016) have been made publicly available via Open Science Framework and can be accessed at https://osf.io/84zg3/.

2 Individualism was also measured. Given the theoretical interest in collectivism, we focused only on collectivism. The pattern of results remained consistent when controlling for individualism.

3 There was a positive correlation between importance of religion and xenophobia without controlling for any variables, *r*(998) = 0.23, *p* < 0.001 (see Table 1). However, once controlled for political ideology, age, gender, income and education, the correlation between importance of religion and xenophobia was not significant, *r*(998) = 0.07, *p* > 0.05 (see Table 2). For discussion on this, see p. 17 (^∗∗^needs to be updated based on the final version). (See Table 1 for bivariate correlations among other variables and Table 2 for partial correlations among key variables controlling for demographic covariates).

4 We also explored an alternative SEM model, switching the order of protection efficacy and collectivism due to not-confirmed directionality between variables, given the correlational nature of the data. Since protection efficacy was measured later and about a more specific context, we expected this alternative model to be a poorer fit. The results confirmed the prediction: comparative fit index (CFI) = 0.71, root-mean-square error of approximation (RMSEA) = 0.11, χ^2^(47) = 575.62, standardized root-mean-square-residual (SRMR) = 0=.07. We explored two other alternative models, which also resulted in poorer fit. For fit indices of these alternative models, please See Supplementary Material.
