# Supplementary material for: Religion, Social Connectedness, and Xenophobic Responses to Ebola
Source: Front Psychol. 2021 Jul 12;12:678141. doi: 10.3389/fpsyg.2021.678141 (PMC8311165; doi:10.3389/fpsyg.2021.678141)
Supplement: Supplementary file 1 [file Table_1.DOCX]

**Religion, Social Connectedness, and Xenophobic Responses to Ebola**

**Supplemental Material**

**SEM Analyses Without Demographic Covariates**

We ran the series of SEM analyses in the main text without demographic covariates: political ideology, gender, age, education, and income.

***Moderation Analysis: Importance of Religion as Moderator***

We examined whether importance of religion moderated the association between perceived vulnerability and xenophobia without any control variables. Higher importance of religion predicted higher xenophobia (at the mean level of perceived vulnerability), *b* = 0.06, *SE* = 0.03, 95% CI of *b* = [0.01, 0.12], *p* = .033. Higher perceived vulnerability significantly predicted greater xenophobia (at the mean level of importance of religion), *b* = 0.45, *SE* = 0.05, 95% CI of *b* = [0.35, 0.54], *p* < .001. More importantly, there was a significant interaction between perceived vulnerability and importance of religion on xenophobia, *b* = -0.10, *SE* = 0.03, 95% CI of *b* = [-0.17, -0.03], *p* = .003. Consistent with the results reported in the main text, higher perceived vulnerability predicted higher xenophobia to a lesser extent among those with higher importance of religion (one standard deviation above the mean), *b* = 0.32, *SE* = 0.06, 95% CI of *b* = [0.21, 0.44], *p* < .001, compared to those with lower importance of religion (one standard deviation below the mean), *b* = 0.57, *SE* = 0.07, 95% CI of *b* = [0.44, 0.70], *p* < .001.

***Mediated Cultural Moderation Analysis: Collectivism as Mediator***

We conducted a mediated cultural moderation analysis to examine whether collectivism explains the moderating effect of importance of religion on the association between perceived vulnerability and xenophobia without covariates. The results are presented in Table S1 below. The key findings reported in the main text (see Table 3) remained consistent.

Table S1.

SEMs examining whether collectivism mediates the moderating effect of importance of religion on the association between vulnerability and xenophobia without demographic covariates.

|  | SEM 1  criterion:  Xenophobia | | SEM 2  criterion:  Collectivism | | SEM 3  criterion:  Xenophobia | |
| --- | --- | --- | --- | --- | --- | --- |
| Predictor | *β* *(b)* | *z* | *β* *(b)* | *z* | *β* *(b)* | *z* |
| Vulnerability | .38 (0.45) | 9.59^***^ | .16 (0.21) | 5.75^***^ | .40 (0.47) | 9.82^***^ |
| Importance of Religion | .08 (0.06) | 2.14^*^ | .44 (0.39) | 15.38^***^ | .08 (0.06) | 1.89 |
| Vulnerability X Religion | -.10 (-0.10) | -2.96^**^ | -.04 (-0.05) | -1.46 | -.06 (-0.06) | -1.62 |
| Collectivism |  |  |  |  | -.01 (-0.01) | -0.33 |
| Vulnerability X Collectivism |  |  |  |  | -.11 (-0.10) | -2.85^**^ |

^*^*p*<.05, ^**^*p*<.01, ^***^*p*<.001

***Mediated Moderation Analysis: Protection Efficacy as Mediator***

We tested whether the vulnerability X importance of religion interaction predicted xenophobia via protection efficacy as mediator without any control variables. Consistent with the results including control variables, protection efficacy partially mediated the interaction between perceived vulnerability and importance of religion on xenophobia. Specifically, the interaction between perceived vulnerability and importance of religion significantly predicted protection efficacy, *b* = 0.10, *SE* = 0.03, 95% CI of *b* = [0.04, 0.17], *p* = .002. The negative association between perceived vulnerability and protection efficacy was weaker among those with higher, relative to lower, importance of religion. Protection efficacy in turn predicted xenophobic responses, *b* = -0.20, *SE* = 0.03, 95% CI of *b* = [-0.26, -0.13], *p* < .001.

The indirect effect of interaction between perceived vulnerability and importance of religion on xenophobia via protection efficacy was significant, *b* = -0.02, *SE* = 0.01, 95% CI of *b* = [-0.04, -0.01], *p* = .005, and the direct effect was also significant, *b* = -0.08, *SE* = 0.03, 95% CI of *b* = [-0.15, -0.02], *p* = .016. In addition, the direct effect of vulnerability on xenophobia was significant, *b* = 0.29, *SE* = 0.05, 95% CI of *b* = [0.19, 0.39], *p* < .001. The direct effect of importance of religion on xenophobia was not significant, *b* = 0.05, *SE* = 0.03, 95% CI of *b* = [-0.01, 0.10], *p* = .082.

***SEM Analysis***

We tested the full model shown in Figure 1 in the main text without any control variables too. The main results remained consistent. Results showed an acceptable fit: comparative fit index (CFI) = .91, root-mean-square error of approximation (RMSEA) = .09, χ^2^(23) = 192.94, standardized root-mean-square-residual (SRMR) = .06. Importance of religion was a significant predictor of collectivism; greater importance of religion predicted higher collectivism, *b* = 0.43, *SE* = 0.02, 95% CI of *b* = [.38, .48], *p* < .001. Collectivism in turn moderated the association between perceived vulnerability and xenophobia via both direct and indirect paths. The direct path of the interaction between vulnerability and collectivism on xenophobia was significant, *b* = -0.08, *SE* = 0.04, 95% CI of *b* = [-0.15, -.011], *p* = .024, as was its indirect path through protection efficacy, *b* = -0.02, *SE* = 0.01, 95% CI of *b* = [-0.04, -0.01], *p* = .003. Lower protection efficacy predicted greater levels of xenophobia, *b* = -0.19, *SE* = 0.03, 95% CI of *b* = [-0.25, -0.12], *p* < .001. The original interaction between vulnerability and importance of religion became non-significant in predicting both protection efficacy and xenophobia in this model in which the interaction between vulnerability and collectivism was included (*b* = 0.06, *SE* = 0.04, 95% CI of *b* = [-0.01, 0.13], *p* = .090 in predicting protection efficacy; *b* = -0.05, *SE* = 0.04, 95% CI of *b* = [-0.12, 0.02], *p* = .181 in predicting xenophobia).

**Mediated Cultural Moderation Analysis with Protection Efficacy as Outcome**

We also ran a mediated cultural moderation analysis with protection efficacy as the outcome variable. Through the series of analysis below, we tested whether importance of religion moderated the association between perceived vulnerability and protection efficacy and whether the moderating effect of importance of religion, if any, was explained (mediated) by collectivism. Consistent with the analyses in the main text, we controlled for political ideology, gender, age, education, and income.

In the first analysis, we found a significant interaction between vulnerability and importance of religion on protection efficacy, *b* = 0.08, *SE* = 0.03, 95% CI of *b* = [0.01, 0.15], *p* = .020. Specifically, the negative slope between vulnerability and efficacy was weaker among those with higher, relative to lower, importance of religion. In the second analysis, we found that higher importance of religion predicted stronger collectivism, *b* = 0.39, *SE* = 0.03, 95% CI of *b* = [0.33, 0.44], *p* < .001. Finally, in the third analysis, the vulnerability X collectivism and the vulnerability X importance of religion interactions were entered simultaneously. While the interaction between vulnerability and collectivism on protection efficacy was significant, *b* = 0.13, *SE* = 0.04, 95% CI of *b* = [0.06, 0.20], *p* < .001, the original interaction between vulnerability and importance of religion became non-significant, *b* = 0.03, *SE* = 0.04, 95% CI of *b* = [-0.04, 0.10], *p* = .405. Thus, it suggests that collectivism fully explains the moderating effect of importance of religion on the association between vulnerability and protection efficacy, consistent with our theoretical model (see Figure 1 in the main text). Specific results from each analysis are presented in Table S2 below.

Table S2.

SEMs examining whether collectivism mediates the moderating effect of importance of religion on the association between vulnerability and protection efficacy.

|  | SEM 1  criterion:  Efficacy | | SEM 2  criterion:  Collectivism | | SEM 3  criterion:  Efficacy | |
| --- | --- | --- | --- | --- | --- | --- |
| Predictor | *β* *(b)* | *z* | *β* *(b)* | *z* | *β* *(b)* | *z* |
| Vulnerability | -.53 (-.76) | -17.93^***^ | .18 (.24) | 5.83^***^ | -.57 (-.82) | -18.77^***^ |
| Importance of Religion | -.04 (-.04) | -1.28 | .43 (.39) | 13.69^***^ | -.07 (-.07) | -2.09^*^ |
| Vulnerability X Religion | .07 (.08) | 2.32^*^ | -.05 (-.06) | -1.73 | .03 (.03) | 0.83 |
| Collectivism |  |  |  |  | .09 (.10) | 2.74^**^ |
| Vulnerability X Collectivism |  |  |  |  | .12 (.13) | 3.71^***^ |

^*^*p*<.05, ^**^*p*<.01, ^***^*p*<.001

**Model Fit Indices of Alternative Models**

***Switching Efficacy and Collectivism (also reported in footnote 4)***

We also explored an alternative SEM model, switching the order of protection efficacy and collectivism due to not-confirmed directionality between variables, given the correlational nature of the data. Since protection efficacy was measured later and about a more specific context, we expected this alternative model to be a poorer fit. The results confirmed the prediction: comparative fit index (CFI) = .71, root-mean-square error of approximation (RMSEA) = .11, χ^2^(47) = 575.62, standardized root-mean-square-residual (SRMR) = .07.

***Switching Collectivism and Importance of Religion***

We explored another alternative SEM model, switching the order of collectivsm and importance of religion due to not-confirmed directionality between variables, given the correlational nature of the data. The results suggested a slightly poorer fit: comparative fit index (CFI) = .83, root-mean-square error of approximation (RMSEA) = .09, χ^2^(48) = 360.48, standardized root-mean-square-residual (SRMR) = .06.

***Switching Efficacy and Xenophobia***

We explored another alternative SEM model, switching the order of protection efficacy and xenophobia due to not-confirmed directionality between variables, given the correlational nature of the data. Since protection efficacy was measured before xenophobia, we expected this alternative model to be a poorer fit. The results confirmed the prediction: comparative fit index (CFI) = .75, root-mean-square error of approximation (RMSEA) = .10, χ^2^(47) = 495.80, standardized root-mean-square-residual (SRMR) = .08.
